# The Warburg effect drives dedifferentiation through epigenetic reprogramming

**DOI:** 10.20892/j.issn.2095-3941.2023.0467

**Published:** 2024-02-05

**Authors:** Haowen Jiang, Mohamed Jedoui, Jiangbin Ye

**Affiliations:** 1 Department of Radiation Oncology, Stanford University School of Medicine, Stanford, CA 94305, USA; 2 Cancer Biology Program, Stanford University School of Medicine, Stanford, CA 94305, USA; 3 Stanford Cancer Institute, Stanford University School of Medicine, Stanford, CA 94305, USA

German biochemist and cell physiologist, Otto H. Warburg (**Figure 1**), made a groundbreaking discovery in 1923. Specifically, tumors were shown to consume large amounts of glucose and ferment glucose into lactate, even in the presence of oxygen—a phenomenon termed “aerobic glycolysis”^1,2^. This phenomenon, later named the Warburg effect by Efraim Racker in the 1970s, remains pivotal in cancer research^3^. Elevated glucose uptake upon Warburg effect induction serves as the foundation for tumor detection in positron emission tomography (PET) scans^4^; however, two critical questions remain unanswered: 1. Why do cancer cells expel lactate rather than utilize lactate for biosynthesis and energy production? 2. How can we strategically target the Warburg effect to treat cancer? Addressing these questions is not only essential for understanding cancer metabolism but also necessary in our quest to find a cure for cancer.

**Figure 1:**
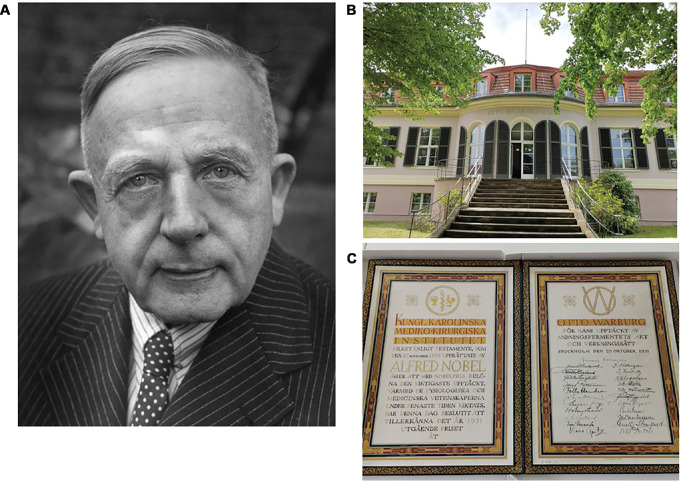
Otto H. Warburg and the Otto-Warburg-Haus. (A) Otto Heinrich Warburg (October 8, 1883–August 1, 1970), German biochemist and cell physiologist, is best known for his discovery of the Warburg effect. The photo is courtesy of Florian Spillert and Susanne Uebele from the Archives of the Max Planck Society. (B) The Otto-Warburg-Haus, originally founded by Warburg in 1930 as the Kaiser Wilhelm Institute for Cell Physiology, underwent a conversion to become the Archive of the Max Planck Society in 1975, after Warburg’s passing. The Archive of the Max Planck Society is located at Boltzmannstraße 14, 14195 Berlin-Dahlem, Germany. (C) Warburg’s Nobel Prize diploma, stored at the Archive of the Max Planck Society. In 1931, Warburg received the Nobel Prize in Physiology as the sole laureate, recognized for his seminal discovery of the nature and mode of action of the respiratory enzyme. (B) and (C) During a personal pilgrimage to Dahlem in May 2023, J.Y. took these photographs.

Warburg proposed that dysfunctional mitochondria are the root cause of the Warburg effect in 1956. Indeed, dysfunctional mitochondria lead to the dedifferentiation of normal terminally differentiated somatic cells, resulting in the formation of tumor cells^5,6^. This revolutionary hypothesis emerged nearly seven decades ago. At that time understanding how metabolic reprogramming influences gene expression and cell fate was limited. Consequently, few researchers and clinicians accepted Warburg’s theory and even fewer sought to validate Warburg’s theory.

In recent years rapid development in the fields of tumor metabolism and epigenetics has gradually allowed us to unravel the century-old enigma of the Warburg effect^7^. This year commemorates the centennial of the Warburg effect discovery. This review aims to encapsulate the present understanding of the Warburg effect and highlights epigenetics as the crucial link between metabolic reprogramming and cell dedifferentiation. Given this connection, activating mitochondrial respiration using mitochondrial uncoupler emerges as a promising therapeutic approach to remodel the cancer epigenome, activate cancer cell differentiation, and potentially cure cancer.

## The Warburg effect: metabolic rewiring

**Figure 2:**
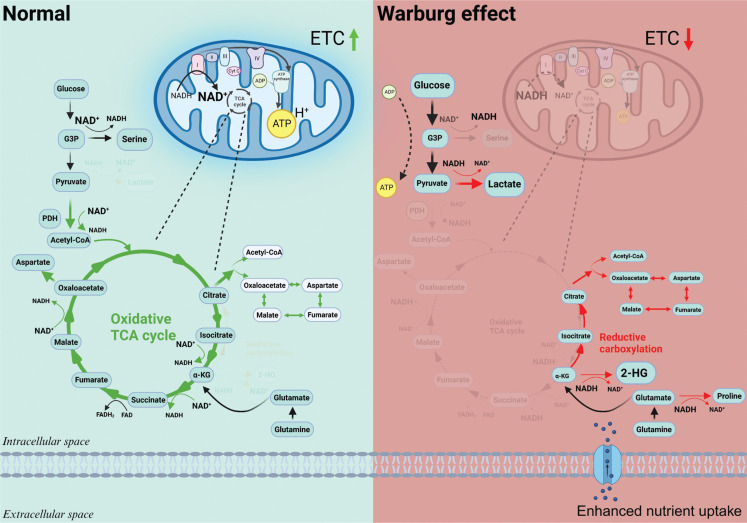
The Warburg effect and metabolic rewiring. The Warburg effect arises due to impaired mitochondrial function, which significantly affects key metabolic processes. Mitochondria, which are crucial for ATP synthesis, NAD^+^ regeneration, the synthesis of macromolecules and epigenetic regulations, become dysfunctional when the electron transport chain (ETC) is inhibited. As a consequence, cells undergo adaptive changes, increasingly depend on glycolysis to produce ATP, modify various pathways for NAD^+^ regeneration, and remodel the epigenome by altering the levels of some metabolites.

The Warburg effect is manifest when mitochondrial function is compromised. Due to the higher mutation rate in mitochondrial DNA compared to nuclear DNA, the accumulation of mitochondrial DNA mutations during the aging process leads to metabolic reprogramming and tumorigenesis^8–10^. Metabolically, mitochondria serve four crucial roles: (a) orchestrate glucose and lipid oxidation, culminating in the establishment of a proton gradient, which is pivotal for ATP synthesis; (b) facilitate the conversion of NADH back to NAD^+^ through the action of complex I, which sustains the tricarboxylic acid (TCA) cycle and enables the transfer of mitochondrial NAD^+^ to the cytosol *via* the malate-aspartate shuttle, and in turn maintains the flux of glycolysis; (c) contribute to macromolecular synthesis, including nucleotide and lipid synthesis, by providing acetyl-CoA and aspartate derived from the TCA cycle^11^; and (d) metabolites from the TCA cycle function as substrates of epigenetic modification enzymes to regulate gene expression and cell fate^7,12^. Therefore, when mitochondria are dysfunctional, the sources of ATP, NAD^+^, nucleotides, and lipids become canonically limited. In response, the cell rewires its metabolism and remodels the epigenome to adapt to stress conditions (**Figure 2**).

First, the cell uses glycolytic flux for ATP generation although the efficiency is much lower (4 mol ATP/mol glucose in aerobic glycolysis *vs*. 36 mol ATP/mol glucose in oxidative phosphorylation)^4^. Additional evidence suggests that the ATP requirement of primary cancer cells is lower when compared to healthy counterparts^13^. Thus, ATP availability does not typically limit the growth of cancer cells. Second, the final product of glycolysis—pyruvate is converted to lactate for regenerating NAD^+^, which is needed for sustaining glycolytic flux^3,14^, even though cancer cells maintain a lower NAD^+^/NADH (a condition known as reductive stress)^15^. This reductive stress drives multiple metabolic pathways, such as the conversion of alpha-ketoglutarate (α-KG) to 2- hydroxyglutarate (HG), reductive carboxylation, and *de novo* proline synthesis^14,16^. Cancer cells exploit these pathways to replenish the NAD^+^ supply. Last but perhaps most important, metabolic reprogramming causes epigenetic remodeling to induce cell dedifferentiation^17^.

## Tumorigenesis is the consequence of dedifferentiation

Differentiation is the intricate process through which pluripotent stem cells within the body divide and specialize. These pluripotent cells acquire distinct structural and functional characteristics, ultimately becoming various somatic cell types. In contrast, dedifferentiation involves regression of normal somatic cells, causing somatic cells to forfeit distinctive morphologic and functional attributes, thereby transforming somatic cells into tumor cells. Tumor grade, a crucial aspect of tumor pathology, denotes the degree to which cancerous cells mirror the characteristics of normal, healthy cells in terms of structure, function, and organization. Well-differentiated tumors closely mimic the tissue from which the tumor originates, often displaying organized cellular patterns and specialized functions. Conversely, poorly differentiated tumors exhibit irregular cellular features and lack clear tissue architecture, making it difficult to pinpoint the source^18^. The differentiation status of a tumor holds paramount importance in patient prognosis and treatment planning. Highly differentiated tumors are generally less aggressive and respond better to therapeutic interventions. Conversely, poorly differentiated tumors often grow rapidly and are linked to a poorer prognosis. Therefore, assessing tumor differentiation is crucial in developing effective treatment plans for patients battling cancer.

## Epigenetics: the missing link between the Warburg effect and tumor dedifferentiation

Epigenetic modifications refer to various changes that regulate gene expression without altering the DNA sequence. More specifically, epigenetic modifications primarily involve DNA methylation and various modifications of histones. All epigenetic modifications share two major characteristics. First, epigenetic modifications are reversible. These dynamic modifications grant cells the plasticity to modulate gene transcription in reaction to a range of signals and stimuli. Second, all epigenetic modifications require small molecule metabolites as substrates^12^. The concentration of metabolites determine the status of various epigenetic modifications, providing a theoretical foundation for the metabolic regulation of cell fate. The majority of metabolites required for epigenetic modifications are derived from the TCA cycle, indicating that mitochondrial respiration plays a pivotal role in controlling epigenetic regulation. This system likely evolved during the process of symbiogenesis, enabling mitochondria to maintain communication and exert a degree of control over nuclear gene expression, even after transferring most of the genome into the nucleus^17^.

Upon mention of the Warburg effect, the first thing that comes to mind is often the production of lactate. It is commonly believed that the substantial production of lactate in tumors is due to overexpression of lactate dehydrogenase (LDH) induced by Myc and hypoxia-inducible factor 1 (HIF-1). However, this could be a misconception because enzymes act as catalysts and only accelerate reactions without changing the ultimate reaction equilibrium. According to the law of mass action, the final equilibrium of a reaction is determined by the product-to-substrate concentration ratio. Therefore, the high production of lactate in tumor cells is likely not attributed to overexpression of LDH, but rather due an excess of NADH in the cells, which drives the conversion of pyruvate-to-lactate. So, why do tumor cells have an excess of NADH? Most of the NADH in the cell is consumed and regenerated to NAD^+^ *via* the mitochondrial electron transport chain (ETC). When mitochondrial respiration is inhibited and the activity of the ETC decreases, the consumption of NADH through oxidation is reduced, leading to increased intracellular NADH levels. The NAD^+^:NADH ratio in most tumor cells is lower than normal cells^15^. Excessive NADH not only converts pyruvate-to-lactate, but also converts α-KG to L-2-hydroxyglutarate (L-2-HG). Like D-2-HG produced by mutant isocitrate dehydrogenase (mIDH), L-2-HG inhibits various dioxygenases that use α-KG as a substrate^19^, including ten-eleven translocation (TET) DNA demethylases, Jumonji domain-containing histone demethylases (JMJDs), and prolyl hydroxylases (PHDs), leading to epigenetic dysregulation (DNA and histone hypermethylation), as well as upregulation of HIF, resulting in cell dedifferentiation^16^.

In addition, multiple metabolic stress conditions inhibit the glucose flux into mitochondria, resulting in lower intracellular acetyl-CoA generation from pyruvate^20,21^. Acetyl-CoA, essential for histone acetylation and crucial for gene transcription and cell differentiation, becomes deficient under metabolic stress conditions. This deficiency inhibits histone acetylation, leading to suppressed tissue differentiation. Under hypoxic conditions, for example, activation of pyruvate dehydrogenase kinases (PDK1/PDK3) *via* HIF signaling of phosphorylate pyruvate dehydrogenase (PDH) leads to a reduction in endogenous acetyl-CoA production, resulting in histone hypoacetylation and subsequently inhibiting the differentiation of neuroblastoma cells. Conversely, supplementation with acetate and other precursors of acetyl-CoA, such as glyceryl triacetate (GTA), restores histone acetylation and promotes cell differentiation, even in hypoxic environments^21^. Another example involves serine deficiency. Serine is an allosteric activator of pyruvate kinase M2 (PKM2)^22^. Serine starvation leads to PKM2 inactivation, which reduces pyruvate and acetyl-CoA production. As a result, histones become hypoacetylated, leading to downregulation of the estrogen receptor (ER) and progesterone receptor (PR). This process transforms ER^+^ PR^+^ breast cancer cells into ER^−^ PR^−^ cells^20^ and renders these cancer cells insensitive to hormone therapy. This finding is consistent with the poorly differentiated breast cancer response to hormone therapy. Intriguingly, serine starvation also leads to downregulation of the mitochondrial citrate transporter, SLC25A1. Reducing the expression of SLC25A1 with shRNA results in decreased histone acetylation and ER expression. Conversely, overexpressing SLC25A1 partially restores histone acetylation and ER expression^20^. This finding suggests that compartmentalization of acetyl-CoA has a crucial role in determining ER expression.

Importantly, serine starvation leads to a reduction in lactate production, demonstrating an “anti-Warburg effect”^20,23^. This anti-Warburg effect indicates that lactate production is not invariably linked to tumor progression. Hypoxia and serine starvation share two common features: 1) decreased pyruvate entering the TCA cycle, leading to reduced acetyl-CoA generation and histone acetylation; and 2) a lower NAD^+^:NADH ratio, reflecting inhibition of the ETC^20,21^. The two central features, which are typically associated with the Warburg effect and tumorigenesis, result in histone hypoacetylation and histone/DNA hypermethylation, which in turn remodels the epigenome and induces cellular dedifferentiation.

## From the Warburg effect to differentiation therapy

PDH is an NAD^+^-dependent enzyme complex. The conversion of pyruvate to acetyl-CoA is governed by the NAD^+^:NADH ratio. Similarly, 3-phosphoglycerate dehydrogenase (PHGDH), the initial enzyme in the *de novo* serine synthesis pathway, is also NAD^+^-dependent^24^. Therefore, the NAD^+^:NADH ratio has a critical role in sustaining the serine synthesis flux, which may be essential for maintaining PKM2 activity and the production of pyruvate. Based on these concepts, inhibition of mitochondrial ETC activity leads to a rise in the NADH concentration. This elevation is the primary cause of the Warburg effect and cell dedifferentiation. Theoretically, activating the mitochondrial ETC to convert NADH back to NAD^+^ reverses the Warburg effect, decreases L-2-HG production, and promotes the differentiation of tumor cells.

**Figure 3:**
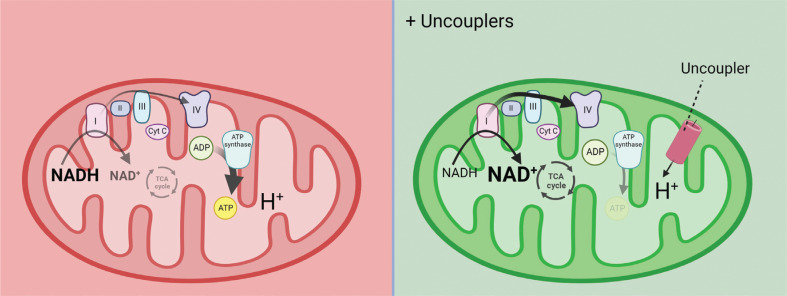
The primary target of mitochondrial uncoupler. The mitochondrial electron transport chain (ETC) generates ATP through a proton gradient formed during substrate oxidation. When ATP synthesis slows down, the proton gradient builds up, inhibiting the ETC and NADH oxidation. To reactivate ETC, it is crucial to decrease the proton gradient. Mitochondrial uncouplers achieve this by dissipating the gradient. **Figures 2** and **3** were generated using BioRender.

How can the ETC be activated? The ETC generates a proton gradient across the mitochondrial inner membrane by oxidizing substrates, such as NADH. ATP synthase uses this proton gradient to synthesize ATP, which provides the energy required for cellular activities and couples ATP synthesis to the ETC. However, when the rate of ATP synthesis decreases and the transmembrane proton gradient increases, the ETC is inhibited, which leads to an accumulation of NADH. Thus, the key to activating the ETC is to reduce the cross-membrane proton gradient in mitochondria. Mitochondrial uncouplers dissipate the proton gradient, thus reactivating the ETC (**Figure 3**).

Niclosamide ethanolamine (NEN) is a promising uncoupling agent with repurposing potential in cancer therapy. NEN is an FDA-approved anti-helminthic drug and is known for low toxicity and a high safety profile^25^. NEN treatment increases the NAD^+^:NADH ratio, indicating activation of the ETC. An increased NAD^+^:NADH ratio^26^ is also associated with increased pyruvate:lactate and α-KG:2-HG ratios, indicating suppression of the Warburg effect and 2-HG production^26^. NEN treatment inhibits reductive carboxylation, an essential pathway that supports tumor cell growth and survival when the ETC is inhibited^27^. Tumor cell proliferation ceases and tumor cells undergo a morphologic transformation into neuron-like cells^26^. Transcriptomic analysis has shown upregulation of genes related to neurodevelopment and neuronal differentiation, while epigenomic analysis demonstrated demethylation in the promoter regions of these genes and increased methylation in gene body regions. We, along with other research groups, have reported that NEN uniquely targets cancer through a systemic approach. NEN activates tumor suppressors, such as p53, AMPK, and PP2A, while concurrently repressing various oncogenic signaling pathways, including Ras, Myc, E2F, Wnt/β-catenin, Notch, mTOR, and Stat3^26,27^. Nevertheless, it would be inaccurate to suggest that the NEN effects are ‘off-target,’ because NEN is not a targeted therapy in the conventional sense. Given the heterogeneity of tumors and the common occurrence of resistance and relapse following targeted therapies, the role of NEN as a mitochondrial uncoupler is unique. NEN exerts control over various pathways by reprogramming metabolism and epigenetics, thereby promoting differentiation and inhibiting tumor growth. This multifaceted approach positions NEN as a promising candidate for tumor therapy.

Based on our studies, we have not only validated the Warburg hypothesis but also established a theoretical framework to interpret the Warburg effect and the mechanisms driving tumorigenesis. This achievement lays a solid foundation for employing mitochondrial uncouplers in tumor differentiation therapy and opens up new avenues for future cancer research and treatment strategies.

## Conclusions and future research

Warburg once said ‘Cancer, above all other diseases, has countless secondary causes. But, even for cancer, there is only one prime cause. Summarized in a few words, the prime cause of cancer is the replacement of the respiration of oxygen in normal body cells by a fermentation of sugar.’ This review illuminates the pivotal role of the Warburg effect in tumorigenesis, particularly the profound impact of the Warburg effect on tumor dedifferentiation. The intricate interplay between dysfunctional mitochondria, epigenetic modifications, and cell fate are being elucidated, providing a straightforward understanding of this century-old enigma. Looking ahead, future research endeavors should delve deeper into the molecular mechanisms underlying the causes of the Warburg effect and explore additional metabolic factors regulating epigenetics to improve differentiation therapy. Clinical validation of promising mitochondrial uncouplers, such as NEN and BAM15, is imperative to translate these findings into clinical practice. The potential synergies between metabolic and differentiation therapy and established treatments, along with a focus on long-term effects and strategies for preventing relapse or resistance through lifestyle intervention, offer exciting avenues for advancing cancer therapeutics.
